# *Aster glehni* Extract Containing Caffeoylquinic Compounds Protects Human Keratinocytes through the TRPV4-PPAR*δ*-AMPK Pathway

**DOI:** 10.1155/2018/9616574

**Published:** 2018-12-09

**Authors:** Yong-Jik Lee, Yoo-Na Jang, Yoon-Mi Han, Hyun-Min Kim, Changbae Jin, Hyoung Ja Kim, Hong Seog Seo

**Affiliations:** ^1^Cardiovascular Center, Korea University Guro Hospital, 148, Gurodong-ro, Guro-gu, Seoul 08308, Republic of Korea; ^2^Department of Medical Science, Korea University College of Medicine (BK21 Plus KUMS Graduate Program), Main building 6F Room 655. 73, Inchon-ro (Anam-dong 5-ga), Seongbuk-gu, Seoul 136-705, Republic of Korea; ^3^Molecular Recognition Research Center, Materials and Life Science Research Division, Korea Institute of Science and Technology, Hwarangno 14 gil 5, Seoul 136-791, Republic of Korea

## Abstract

*Aster glehni *(AG) has been used in cooking and as a medicine to treat various diseases for over hundreds of years in Korea. To speculate the protective effects of AG on skin barrier, we estimated the protein levels of biomarkers related to skin barrier protection in human keratinocytes, HaCaT cells treated with sodium dodecyl sulfate (SDS), or 2,4-dinitrochlorobenzene (DNCB). The protein levels for keratin, involucrin, defensin, tumor necrosis factor alpha (TNF*α*), peroxisome proliferator-activated receptor delta (PPAR*δ*), 5′ adenosine monophosphate-activated protein kinase (AMPK), serine palmitoyltransferase long chain base subunit 2 (SPTLC2), and transient receptor potential cation channel subfamily V member 4 (TRPV4) were evaluated using western blotting or immunocytochemistry in HaCaT cells. AG extract increased the protein levels of PPAR*δ*, phosphorylated AMPK, SPTLC2, keratin, involucrin, and defensin compared to the SDS or DNCB control group. However, TNF*α* expression increased by SDS or DNCB was decreased with AG extract. The order of action of each regulatory biomarker in AG pathway was identified TRPV4→PPAR*δ*→AMPK from antagonist and siRNA treatment studies. AG can ameliorate the injury of keratinocytes caused by SDS or DNCB through the sequential regulation of TRPV4→PPAR*δ*→AMPK pathway.

## 1. Introduction

The skin, also called integument, consists of three major layers, namely epidermis, dermis, and hypodermis (subcutis). In particular, the epidermis is the outer region of the skin and has four layers—stratum basale, stratum spinosum, stratum granulosum, and stratum corneum (the outermost thin layer of the skin). Keratinocytes are the main cells that make up the epidermis, and they contribute to the construction of a defense wall for the body through keratinization. In stratum spinosum, keratinocytes produce keratin1 and 10, and keratin10 is a main constituent of desmosome. In general, the permeability barrier of skin is provided by stratum corneum and is composed of corneocytes, cornified envelope, corneodesmosome, and intercorneocyte lipid. The cornified envelope comprises transglutaminase, involucrin, loricrin, and filaggrin, and it serves as a complete permeability barrier through the formation of the multilayered structure of intercorneocyte lipid. Interconeocyte lipids comprise ceramide, cholesterol, free fatty acids, and cholesterol sulfate, and, among them, ceramide content is the highest. The main functions of the skin are to prevent the loss of body fluids, attack of toxins, and invasion of pathogens [1–3]. Therefore, the impairment of skin permeability barrier function is not a simple mechanical destruction of the outer layer of the skin. The damaged skin can cause severe immune reactions through the invasion and colonization of various pathogens. Sodium dodecyl sulfate (SDS) is generally known as a skin irritant as well as an anionic surfactant. As a detergent, SDS induces skin surface polarity and skin barrier disruption and can facilitate epidermal lipid extraction [4]. In addition, SDS elevates transepidermal water loss from the stratum corneum and induces inflammation [5, 6].

Although atopic dermatitis (AD) develops in early infancy, it is also seen in adults. In general, AD is a chronic and pruritic inflammatory skin disease characterized by increased immunoglobulin E (IgE) production, relapsing eczematous skin lesions, infiltration of inflammatory immune cells, and defective skin barrier, and its underlying cause is almost unknown [7, 8]. With the advancement of industrialization, AD has become more prevalent. Therefore, finding effective remedies for AD is very urgent and socioeconomically important. 2,4-Dinitrochlorobenzene (DNCB) is reported to induce contact dermatitis and AD [9, 10].

*Aster glehni* (AG) has been used as an edible food, and medicine for over hundreds of years in Korea.

In Korean traditional medicine,* Aster glehni* (AG) has been used to cure fever, pain, phlegm, and cough. Other functions of AG have been reported previously as follows. An ethyl acetate extract of AG inhibited the protein expression of tyrosinase and tyrosinase-related protein 1, which are involved in melanin biosynthesis in melanocytes [11]. In another study, the ethyl acetate extract of AG showed antioxidant effect and inhibited the protein expression of induced nitric oxide synthase (iNOS), which is involved in inflammation [12].

To clarify the effects and mechanism of AG on skin permeability barrier protection, we estimated the expression levels of biomarkers related to permeability barrier maintenance in HaCaT cells treated with SDS or DNCB which is a skin irritant or an inducer of dermatitis containing atopic dermatitis. Particularly, in previous studies, peroxisome proliferator-activated receptor *δ* (PPAR*δ*), AMP-activated protein kinase (AMPK), and transient receptor potential cation channel subfamily V member 4 (TRPV4) participated in the maintaining of structural integrity of keratinocytes [13–17], and in general the impairment of skin permeability barrier mainly composed by keratinocytes induces dermatitis containing atopic dermatitis [18, 19]. Therefore this study was conducted with a focus on the roles of PPAR*δ*, AMPK, and TRPV4 as the regulators in the functions of AG.

## 2. Materials and Methods

### 2.1. Plant Materials, Extraction Procedure, and High-Performance Liquid Chromatography (HPLC) Analysis

Plant materials used in this experiments were parboiled and dried* Aster glehni* F. Schmidt (Compositae), and the extraction procedure and high-performance liquid chromatography (HPLC) analysis referred to our already published paper [20]. 

To acquire a methanol-soluble extract, 12 kg chopped AG were extracted three times with 70 L methanol at room temperature. The dried extract residue of 2.6 kg was suspended in water and then partitioned successively with ethyl acetate. The ethyl acetate extract of AG was analyzed by reverse-phase high-performance liquid chromatography (HPLC) (Waters 1500 Series System), with a 2996 PDA Detector (254 nm, Waters, Worcester, MA, USA). Separation was performed using a Luna C18 column (5 *μ*m, 250 × 4.6 mm, Phenomenex, Torrance, CA, USA) with a sample injection volume of 10 *μ*L at 30°C. The mobile phase was a gradient of acetonitrile and 1% phosphoric acid. The gradient system was as follows: 20% acetonitrile (0 min), 20% acetonitrile (0~10 min), 30% acetonitrile (10~20 min), 40% acetonitrile (20~30 min), 80% acetonitrile (30~40 min), and 100% acetonitrile (40~50 min). The flow rate of the mobile phase was 1.0 ml/min. Organic solvents used in extraction procedure and HPLC analysis were purchased from Sigma-Aldrich (St. Louis, MO, USA).

### 2.2. Cell Culture

HaCaT cells and human keratinocytes were cultured in Dulbecco's modified Eagle's medium (DMEM) containing 10% fetal bovine serum (FBS) and 1% antibiotic-antimycotic solution (AA) in 37°C and 5% CO_2_ incubator. The cell culture medium was replaced with new DMEM every 48-72 h. HaCaT cells within 5~17 passages were plated at a density of 1×10^4^ cells per well in 8-well chamber slide, or 1×10^6^ cells per well in a six-well culture plate in DMEM containing 10% FBS and 1% AA. The cells were cultured for 24 to 48 h in 5% CO_2_ incubator at 37°C. Then, the medium was changed to DMEM containing 1% FBS. Thereafter, the cells were treated by DNCB (5 *μ*mol; Sigma-Aldrich, Louis, MO, USA) or SDS (30 *μ*mol; Sigma-Aldrich) along with AG extract (50 *μ*g) and PPAR*δ* antagonist, GSK0660 (50 *μ*mol; Sigma-Aldrich), AMPK antagonist, Compound C (1 *μ*mol; Sigma-Aldrich), or TRPV4 antagonist, GSK2193874 (50 nmole; Sigma-Aldrich) for 24 hours. HaCaT cells were donated from Dr. Sang-Wook Son. All reagents for cell culture were purchased from WELGENE Inc. (Daegu, Republic of Korea). The treating concentrations of all reagents were final concentrations.

### 2.3. Immunocytochemistry (ICC)

The cells in chamber slide were fixed by ice-cold methanol for 15 min. The fixed cells reacted with 0.3% hydrogen peroxide (H_2_O_2_) solution containing 0.3% normal horse serum for 5 min to remove peroxidase activity in cells. After washing with phosphate-buffered saline (PBS) for 5 min, the cells reacted with diluted normal blocking serum for 20 min, followed by treatment with diluted primary antibodies for 1 h. The primary antibodies for involucrin and TNF*α* were obtained from Novus (Littleton, CO 80120, USA). The primary antibody for defensin*β*1 was purchased from Santa Cruz Biotechnology, Inc. (Dallas, Texas, USA) and that for pan-keratin was purchased from Abcam (Cambridge, UK). After washing with PBS, the cells were treated with secondary antibody for 30 min. After PBS washing, the cells were treated with premixed VECTASTATIN ABC reagent solution for 30 min. The cells were washed with PBS and allowed to react with DAB substrate solution until proper color change was observed. After washing with tap water for 3 min, the cells were counterstained with hematoxylin. The cells were washed with tap water, air-dried, and finally mounted. The number of each group was three every experiments. All experiments were repeated over three times. The validity of ICC results was proved by the comparison with negative control. The immunocytochemistry kit (containing secondary antibody) was purchased from Vector laboratories (Burlingame, CA, USA).

### 2.4. Western Blot Analysis

Protein concentration in sample was estimated by the Bradford method. The extracted proteins (10 *μ*g) were loaded onto 10% SDS-polyacrylamide gels, and protein blotting on nitrocellulose membranes was performed for 90 min. The membranes were blocked overnight with 5% skim milk and washed three times for 10 min with tris-buffered saline containing 0.05% tween 20 (TBS-T). The primary antibodies were allowed to bind to the membranes at room temperature for 2 h. The primary antibody for PPAR*δ* was supplied by Abcam. The primary antibodies for total and phosphorylated forms of AMPK (p-AMPK), and cleaved poly-ADP-ribose polymerase (cPARP) were purchased from Cell Signaling Technology, Inc. (Danvers, MA, USA). The primary antibody for serine palmitoyltransferase 2 (SPTLC 2) was purchased from Novus and that for *β*-actin was procured from Santa Cruz Biotechnology, Inc. The dilution conditions for primary antibodies were as follows: PPAR*δ*, 1:500; AMPK, p-AMPK (at Thr172), cPARP, and SPTLC 2, 1:1000; and *β*-actin, 1:800. After washing three times with TBS-T for 10 min, the membranes were incubated with secondary antibodies (Santa Cruz Biotechnology, Inc.) at room temperature for 1 h. The dilution conditions for secondary antibodies were as follows: anti-rabbit IgG antibodies for PPAR*δ*, AMPK, p-AMPK, cPARP, and SPTLC 2, 1:5000 and anti-mouse IgG antibody for *β*-actin, 1:5000. After three washes with TBS-T for 10 min and a single TBS wash for another 10 min, chemiluminescent substrate and enhancer solution (Bio-Rad, Hercules, CA, USA) were applied to the membranes to determine the protein expression rate. Images were processed manually with Kodak GBX developer and fixer reagents (CARESTREAM HEALTH, INC., Rochester, NY, USA) and analyzed using ImageJ program. The number of each group was three every experiments. All experiments were repeated over three times. *β*-Actin was used as an internal control to normalize the loaded proteins.

### 2.5. Semiquantitative Reverse Transcription Polymerase Chain Reaction (RT-PCR)

Total RNA was extracted via TRIzol® reagent according to the manufacturer's instructions. Complementary DNA was synthesized from total RNA using the Power cDNA Synthesis kit, and polymerase chain reactions for keratin, involucrin, defensin*β*1, TNF*α*, TRPV4, PPAR*δ*, AMPK*α*1, and *β*-actin were performed using PCR Premix kits. The primer sequences used were as follows: forward 5'-CCCTTCTTTCGAGGTCTTGG-3' and reward 5'-TGCTGGAAGGTAGGCACAAG-3' for human keratin (product size- 239 base pairs); forward 5'- CACCTAGAGGAGCAGGAGGG-3' and reward 5'-CAGGTGCTTTGGCTGTCCTA-3' for human involucrin (product size- 216 base pairs); forward 5'- TGAGATGGCCTCAGGTGGTA-3' and reward 5'-AGCTCACTTGCAGCACTTGG-3' for human defensin*β*1 (product size- 163 base pairs); forward 5'- CCCTCAACCTCTTCTGGCTC-3' and reward 5'-AGCTGTAGGCCCCAGTGAGT-3' for human TNF*α* (product size- 187 base pairs); forward 5'- GTTCATGAAGAAATGCCCTG-3' and reward 5'-GTGAGGATGATGTAGGTCAC-3' for human TRPV4 (product size- 522 base pairs); forward 5'- AAGGCCTTCTCCAAGCACAT-3' and reward 5'-AAGACGTGCACGCTGATCTC-3' for human PPAR*δ* (product size- 212 base pairs); forward 5'- ACAAGTTGTGGCTCACCCAA-3' and reward 5'-TGGCACATGGTCATCATCAA-3' for human AMPK*α*1 (product size- 147 base pairs); forward 5'- GCTTGGTCACTTCGTGGCTA-3' and reward 5'-CAAACCGCTTCCAACTCAAA-3' for human *β*-actin (product size- 281 base pairs). The reaction mixture containing cDNA was preheated for 5 minutes at 95°C as an initial denaturation step. Polymerase chain reaction consisted of denaturation step for 20 seconds at 95°C, annealing step for 10 seconds at 55°C, extension step for 30 seconds at 72°C, and final extension step for 5 minutes at 72°C. The number of each group was three every experiment. All experiments were repeated over three times. The normalization for RT-PCR results were done with *β*-actin and GAPDH. TRIzol® reagent was purchased from Invitrogen (Grand Island, NY, USA). Power cDNA Synthesis kit, PCR premix, and DNA ladder (100 base pair) were bought from Intron Biotechnology (Seongnam-si, Gyeonggi-do, Korea)

### 2.6. Small Interfering RNA (siRNA) Transfection

siRNA solutions for TRPV4, PPAR*δ*, and AMPK*α*1/2 were prepared as follows: solution A is composed of 6 ul siRNA plus 100 ul transfection medium (DMEM having no FBS and AA, WELGENE Inc.) per well, and solution B is composed of 6 ul transfection reagent plus 100 ul transfection medium per well. The mixture of solutions A and B was stirred thoroughly and then incubated for 30 min at room temperature. The incubated mixture was added to cells, the total volume was 1 ml together with medium, and the cells treated with siRNA were incubated for 6 hr in CO_2_ incubator at 37°C. After incubation in CO_2_ incubator, 1 ml DMEM containing 20% FBS was added to the well. siRNAs and transfection reagent were purchased from Santa Cruz Biotechnology.

The sequences for siRNAs are as follows. The human TRPV4 siRNA is a pool of 3 different siRNA duplexes: the sequences of first duplex are sense 5'-CCUUCCGUGACAUCUACUATT-3' and antisense 5'-UAGUAGAUGUCACGGAAGGTT-3'; the sequences of second duplex are sense 5'- GAGAACACCAAGUUUGUUATT-3' and antisense 5'-UAACAAACUUGGUGUUCUCTT-3'; the sequences of third duplex are sense 5'-CUGCUCUACUUCAUCUACUTT-3' and antisense 5'- AGUAGAUGAAGUAGAGCAGTT -3'. The human PPAR*δ* siRNA is a pool of 3 different siRNA duplexes: the sequences of first duplex are sense 5'-GGUUACCCUUCUCAAGUAUTT-3' and antisense 5'-AUACUUGAGAAGGGUAACCTT-3'; the sequences of second duplex are sense 5'- CCUUCAGUGAUAUCAUUGATT-3' and antisense 5'-UCAAUGAUAUCACUGAAGGTT-3'; the sequences of third duplex are sense 5'-CUCCUGUCUUCAGAGCAAATT-3' and antisense 5'- UUUGCUCUGAAGACAGGAGTT-3'. The human AMPK*α*1/2 siRNA is a pool of 3 different siRNA duplexes: the sequences of first duplex are sense 5'-GAUGUCAGAUGGUGAAUUUTT-3' and antisense 5'-AAAUUCACCAUCUGACAUCTT-3'; the sequences of second duplex are sense 5'- CCACUGCAAUACUAAUUGATT-3' and antisense 5'-UCAAUUAGUAUUGCAGUGGTT-3'; the sequences of third duplex are sense 5'-CUACUGGAUUUCCGUAGUATT-3' and antisense 5'- UACUACGGAAAUCCAGUAGTT-3'. The number of each group was three every experiment. All experiments were repeated over three times. The experimental validity was supported by control siRNA treatment group.

### 2.7. Statistics

Data are presented as mean ± SE (Standard Error of Measures). Statistically significant differences between two groups were calculated by the unpaired* t*-test, and one-way ANOVA test was used to compare means of three or more groups with GraphPad Prism software. A value of* p*<0.05 was considered significant.

## 3. Results

### 3.1. The Ethyl Acetate Fraction Extracted from Aster glehni Consists of Caffeoylquinic Compounds of Seven Kinds Mainly

**Figure 1:**
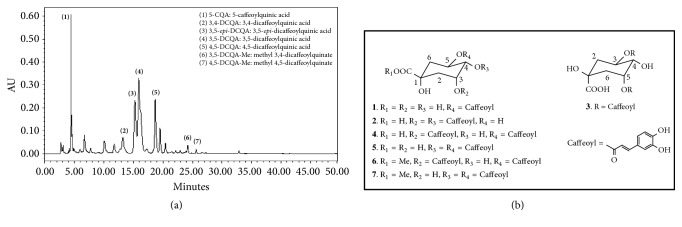
**HPLC analysis chromatogram (a) and the structures (b) of constituent chemicals for* Aster glehni* ethyl acetate extract.** The main phytochemicals identified from* Aster glehni *are caffeoylquinic acids of seven kinds: (1) 5-CQA: 5-caffeoylquinic acid, (2) 3,4-DCQA: 3,4-dicaffeoylquinic acid, (3) 3,5-*epi*-DCQA: 3,5-*epi*-dicaffeoylquinic acid, (4) 3,5-DCQA: 3,5-dicaffeoylquinic acid, (5) 4,5-DCQA: 4,5-dicaffeoylquinic acid, (6) 3,5-DCQA-Me: methyl 3,4-dicaffeoylquinic acid, and (7) 4,5-DCQA-Me: methyl 4,5-dicaffeoylquinic acid.

In our previously published paper, the caffeoylquinic compounds of six kinds in AG were discovered by HPLC analysis [20]. But, through further analysis, it was confirmed that the ethyl acetate fraction extracted from AG mainly consists of caffeoylquinic compounds of seven kinds which consisted of 5-caffeoylquinic acid, 3,4-dicaffeoylquinic acid, 3,5-*epi*-dicaffeoylquinic acid, 3,5-dicaffeoylquinic acid (3,5-DCQA), 4,5-dicaffeoylquinic acid, methyl 3,4-dicaffeoylquinate, and methyl 4,5-dicaffeoylquinate (Figure 1). The 3,5-*epi*-dicaffeoylquinic acid was newly discovered in further HPLC analysis. 3,5-DCQA was the most abundant in the seven caffeoylquinic compounds of the AG ethyl acetate fraction.

### 3.2. Aster glehni Extract Increased the Protein Levels for PPAR*δ*, AMPK, SPTLC2, and TRPV4 of HaCaT Cells in the Condition of DNCB or SDS Treatment

**Figure 2:**
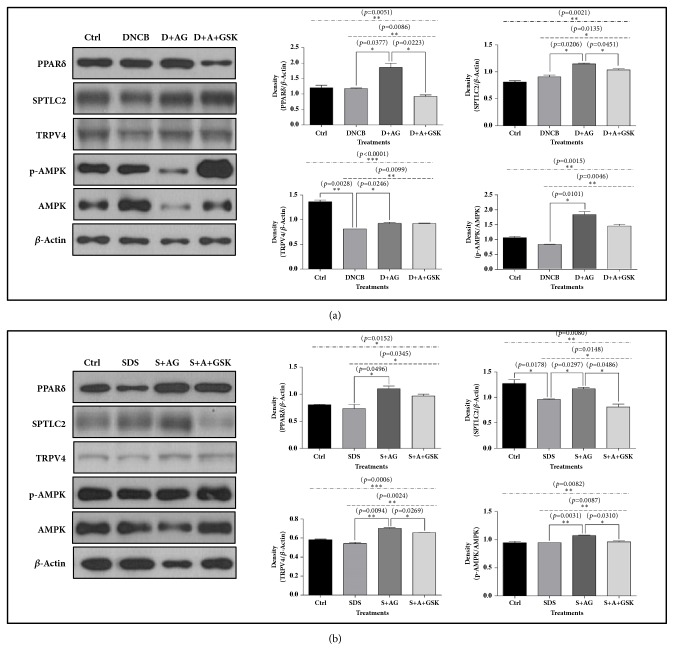
**Western blot analyses for PPAR** **δ** **, AMPK, and SPTLC2 in HaCaT cells treated with* Aster glehni *extract, SDS, DNCB, and GSK0660.** AG increased the protein levels of biomarkers related to the protection of keratinocytes in DNCB (a) or SDS (b) treated condition. At large, the effects of AG were offset by PPAR*δ* antagonist treatment. The results are expressed as means ± SEM. N=3 for each group. Values were statistically analyzed by unpaired* t*-test and one-way ANOVA. All experiments were repeated over three times. The dash-dotted line means the one-way ANOVA result between ctrl and all other groups. The dotted line means the one-way ANOVA result among all groups except for control. Meaning of indications: Ctrl is an untreated control group, DNCB is a DNCB treated group, D+AG is a DNCB+AG treated group, D+A+GSK is a DNCB+AG+GSK0660 treated group, SDS is a SDS-treated group, S+AG is a SDS+AG treated group, and S+A+GSK is a SDS+AG+GSK0660 treated group.

Previous studies reported that PPAR*δ* and AMPK are involved in cell survival and anti-inflammatory response [21–24]. Ceramide is an important and main lipid constituent in skin barrier, and serine palmitoyltransferase is an enzyme catalyzing a rate limiting step in ceramide biosynthesis. In addition, TRPV4 is known to involve in the formation of intercellular junction in keratinocytes [25]. SPTLC2 is a long chain base subunit of serine palmitoyltransferase. Therefore, we estimated the protein levels of PPAR*δ*, AMPK, and SPTLC2 in HaCaT cells treated with SDS or DNCB along with AG. AG extract increased the protein levels for PPAR*δ*, p-AMPK, SPTLC2, and TRPV4 compared to DNCB- and SDS-treated groups. However, the elevated protein levels were reversed by the PPAR*δ* antagonist GSK0660 (Figure 2).

### 3.3. Aster glehni Extract Increased the Protein and mRNA Expressions for Keratin, Involucrin, and Defensin*β*1 of HaCaT Cells in Pathological Conditions; however, TNF*α* Protein and mRNA Expressions Were Decreased by the Extract. The mRNA Levels for TRPV4, PPAR*δ*, and AMPK*α*1 Were also Increased by AG Treatment

Defensins are antimicrobial, antifungal, and antiviral small cationic peptides produced by different cell types, and they include three subfamilies of *α*-, *β*-, and *θ*-defensins. In keratinocytes, *β*-defensins are the mainly secreted subtypes [26]. Therefore, in this study, we evaluated the expression of defensin*β*1, a representative defensin*β*, together with skin barrier constituents such as keratin and involucrin and inflammatory cytokine TNF*α*.

**Figure 3:**
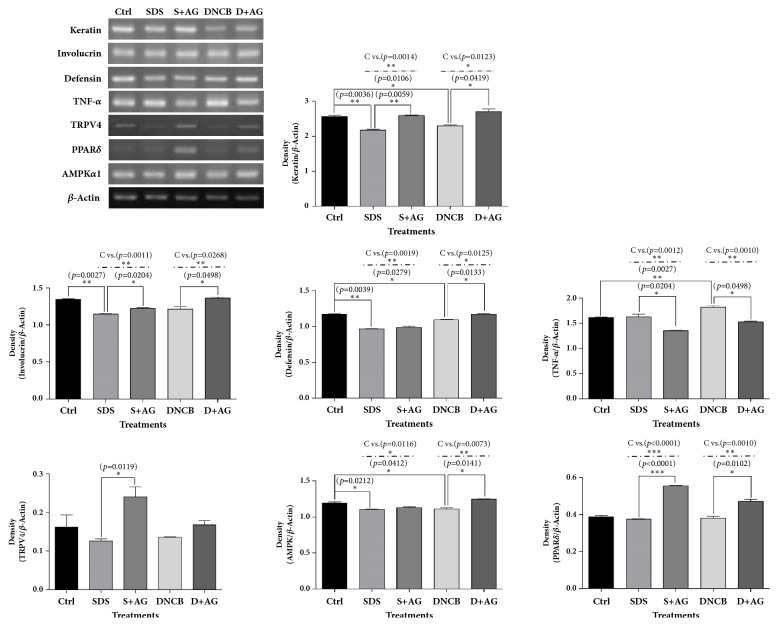
**RT-PCR for keratin, involucrin, defensinn, TNF** **α** **, TRPV4, PPAR** **δ** **, and AMPK in HaCaT cells treated with* Aster glehni *extract, SDS, and DNCB.** AG generally increased the mRNA levels of biomarkers related to the protection of keratinocytes in SDS or DNCB treated condition. But the mRNA level of TNF*α* was decreased by AG treatment. The results are expressed as means ± SEM. N=3 for each group. Values were statistically analyzed by unpaired* t*-test and one-way ANOVA. All experiments were repeated over three times. The dash-dotted line means the one-way ANOVA result between ctrl and other groups. Meaning of indications: Ctrl is an untreated control group, DNCB is a DNCB treated group, D+AG is a DNCB+AG treated group, SDS is a SDS-treated group, and S+AG is a SDS+AG treated group.

**Figure 4:**
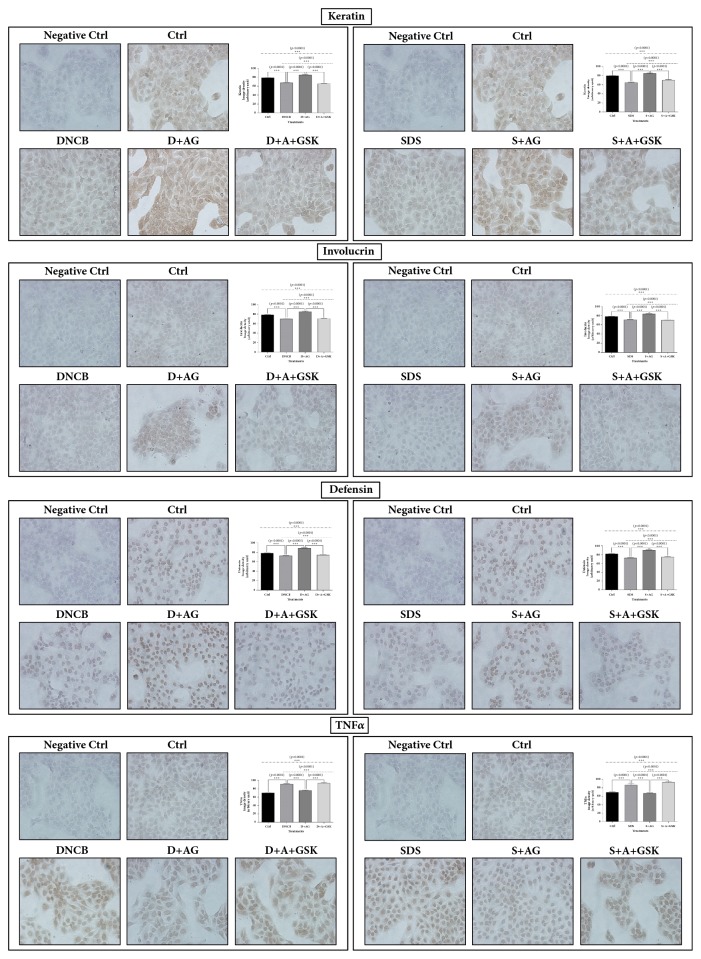
**Immunocytochemistry for keratin, involucrin, defensin, and TNF** **α** **in HaCaT cells treated with* Aster glehni *extract, SDS, DNCB, and GSK0660.** The abnormal expressions for keratin, involucrin, defensin, and TNF*α* by SDS or DNCB treatment were normalized with AG treatment; however, the effects of AG were offset by PPAR*δ* antagonist. Images were taken at 200 × magnification. Densities for images were analyzed with Image J program. The results are expressed as means ± SEM. N=3 for each group. Values were statistically analyzed by unpaired* t*-test and one-way ANOVA. All experiments were repeated over three times. The dash-dotted line means the one-way ANOVA result between ctrl and all other groups. The dotted line means the one-way ANOVA result between all groups except for control. Meaning of indications: Ctrl is an untreated control group, DNCB is a DNCB treated group, D+AG is a DNCB+AG treated group, D+A+GSK is a DNCB+AG+GSK0660 treated group, SDS is a SDS-treated group, S+AG is a SDS+AG treated group, and S+A+GSK is a SDS+AG+GSK0660 treated group.

AG extract increased generally the mRNA levels of keratin, involucrin, defensin*β*1, TRPV4, PPAR*δ*, and AMPK*α*1 related to the protection of keratinocytes in SDS or DNCB treated condition. But the mRNA level of TNF*α* was decreased by AG treatment (Figure 3). AG extract increased the protein expressions of keratin, involucrin, and defensin*β*1, which were decreased by DNCB and SDS; however, the elevated protein levels were offset by the PPAR*δ* antagonist GSK0660. TNF*α* expressions increased by DNCB and SDS and were decreased by AG extract, but the ameliorating effect of AG extract was reversed by GSK0660 (Figure 4).

### 3.4. The mRNA and Protein Expressions of Keratin, Involucrin, and Defensin*β*1 in HaCaT Cells Were Decreased by Antagonists for TRPV4 and AMPK. However, TNF*α* Protein and mRNA Expressions Were Increased

To investigate the effects of TRPV4 and AMPK on the expressions of biomarkers related to skin permeability barrier constituents, defense, and inflammation, the expressions for keratin, involucrin, defensin*β*1, and TNF*α* were evaluated via RT-PCR and immunocytochemistry in HaCaT cells treated with antagonists for TRPV4 and AMPK.

**Figure 5:**
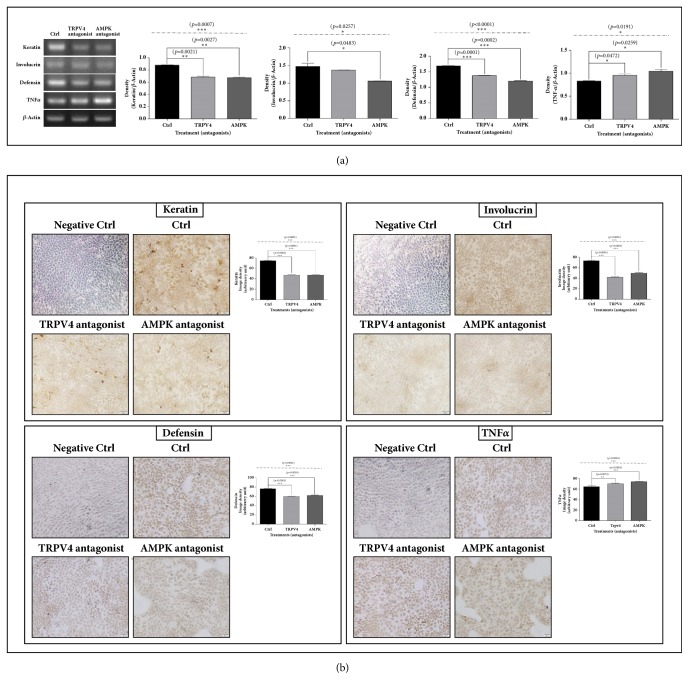
**RT-PCR and ICC results for keratin, involucrin, defensin, and TNF** **α** **in HaCaT cells treated with antagonists for TRPV4 and AMPK.** The antagonists for TRPV4 and AMPK decreased mRNA (a) and protein (b) expressions of keratin, involucrin, and defensin, but they increased TNF*α* expression (a, b). Images were taken at 200 × magnification. The results are expressed as means ± SEM. N=3 for each group. Values were statistically analyzed by unpaired* t*-test and one-way ANOVA. All experiments were over repeated three times. The dash-dotted line means the one-way ANOVA result between ctrl and other groups.

The mRNA and protein levels for TNF*α* were elevated by TRPV4 antagonist or AMPK antagonist compared to the control in HaCaT cells. However, under the same condition, the mRNA and protein expressions of keratin, involucrin, and defensin*β*1 markedly decreased compared to the control (Figure 5).

### 3.5. The mRNA and Protein Expressions of TRPV4, PPAR*δ*, and AMPK*α*1 in HaCaT Cells Were Differently Changed by Antagonists and siRNAs for TRPV4, PPAR*δ*, and AMPK. The mRNA Expressions of TRPV4, PPAR*δ*, and AMPK Increased by AG in HaCaT Cells in Pathological Conditions Were Decreased in TRPV4 Antagonist Treated Group

To determine the priority of major regulators which are TRPV4, PPAR*δ*, and AMPK, we estimated the mRNA and protein levels of TRPV4, PPAR*δ*, and AMPK in HaCaT cells treated with siRNAs and antagonists for TRPV4, PPAR*δ*, or AMPK.

**Figure 6:**
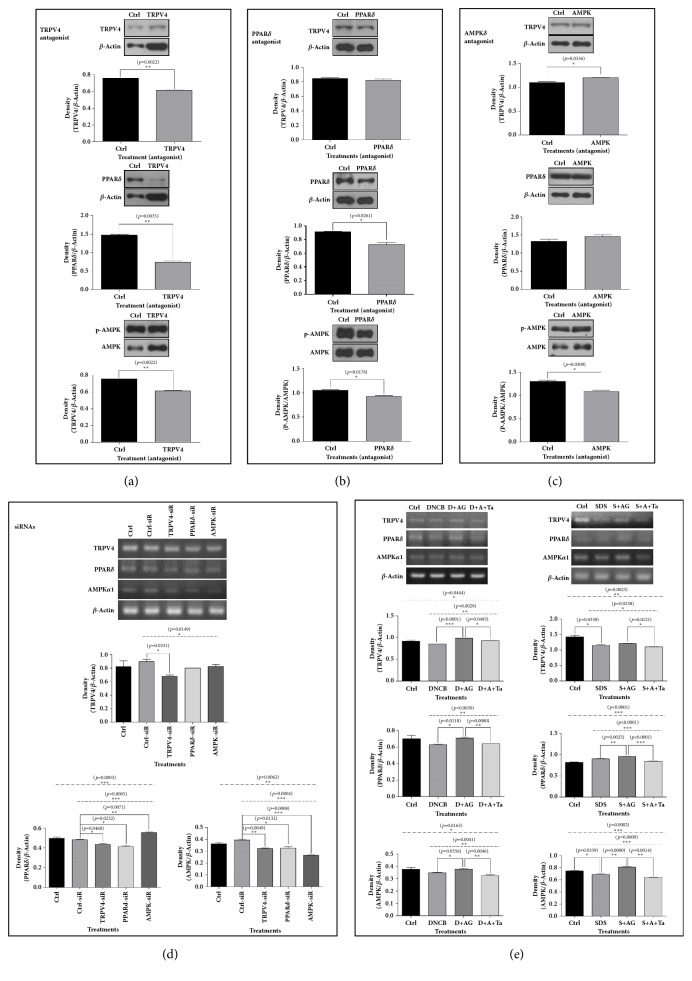
**Western blot analyses and RT-PCR for TRPV4, AMPK, and PPAR** **δ** **in HaCaT cells treated with antagonists or siRNAs for TRPV4, PPAR** **δ** **, and AMPK. RT-PCR for TRPV4, AMPK, and PPAR** **δ** **in HaCaT cells treated with TRPV4 antagonist in pathological conditions.** TRPV4 antagonist and siRNA decreased the protein levels of TRPV4, PPAR*δ*, and AMPK (a, d). PPAR*δ* antagonist and siRNA decreased the protein levels PPAR*δ* and AMPK (b, d). AMPK antagonist and siRNA decreased only AMPK (c, d). In pathological conditions, the TRPV4 antagonist reversed the AG effects on TRPV4, PPAR*δ*, and AMPK (e). The results are expressed as means ± SEM. N=3 for each group. Values were statistically analyzed by unpaired* t*-test and one-way ANOVA. All experiments were repeated over three times. The dash-dotted line means the one-way ANOVA result between ctrl and all other groups. The dotted line means the one-way ANOVA result among groups except for control. Meaning of indications: Ctrl is an untreated control group, DNCB is a DNCB treated group, D+AG is a DNCB+AG treated group, D+A+Ta is a DNCB+AG+TRPV4 antagonist treated group, SDS is a SDS-treated group, S+AG is a SDS+AG treated group, and S+A+Ta is a SDS+AG+TRPV4 antagonist treated group.

In HaCaT cells treated with TRPV4 antagonist, all the protein levels for TRPV4, PPAR*δ*, and p-AMPK were significantly decreased (Figure 6(a)). PPAR*δ* antagonist treatment lowered the protein levels for PPAR*δ* and p-AMPK; however, it did not change the TRPV4 protein expression (Figure 6(b)). The AMPK antagonist, Compound C, decreased the protein expression for only p-AMPK, but it did not affect the protein expressions for TRPV4 and PPAR*δ* (Figure 6(c)). The results of the siRNA experiments were identical to those of the antagonists. TRPV4 siRNA treatment decreased all the mRNA levels for TRPV4, PPAR, and AMPK*α*1 compared to control siRNA treated group. And PPAR*δ* siRNA decreased mRNA levels for PPAR and AMPK*α*1. But AMPK*α*1/2 siRNA lowered only AMPK mRNA expression (Figure 6(d)). In pathological conditions of HaCaT cells induced by DNCB and SDS treatments, the mRNA levels for TRPV4, PPAR*δ*, and AMPK were decreased compared to control group. But the mRNA expressions of TRPV4, PPAR*δ*, and AMPK lowered in the pathological conditions were increased by AG treatment. But the increasing effects of AG on the mRNA expressions for TRPV4, PPAR*δ*, and AMPK in pathological conditions were offset by TRPV4 antagonist treatment (Figure 6(e)).

### 3.6. 3, 5-DCQA Increased the Protein Levels for TRPV4, PPAR*δ*, AMPK, SPTLC2, Keratin, Involucrin, and Defensin*β*1 of HaCaT Cells. However, TNF*α* Expression Was Decreased

**Figure 7:**
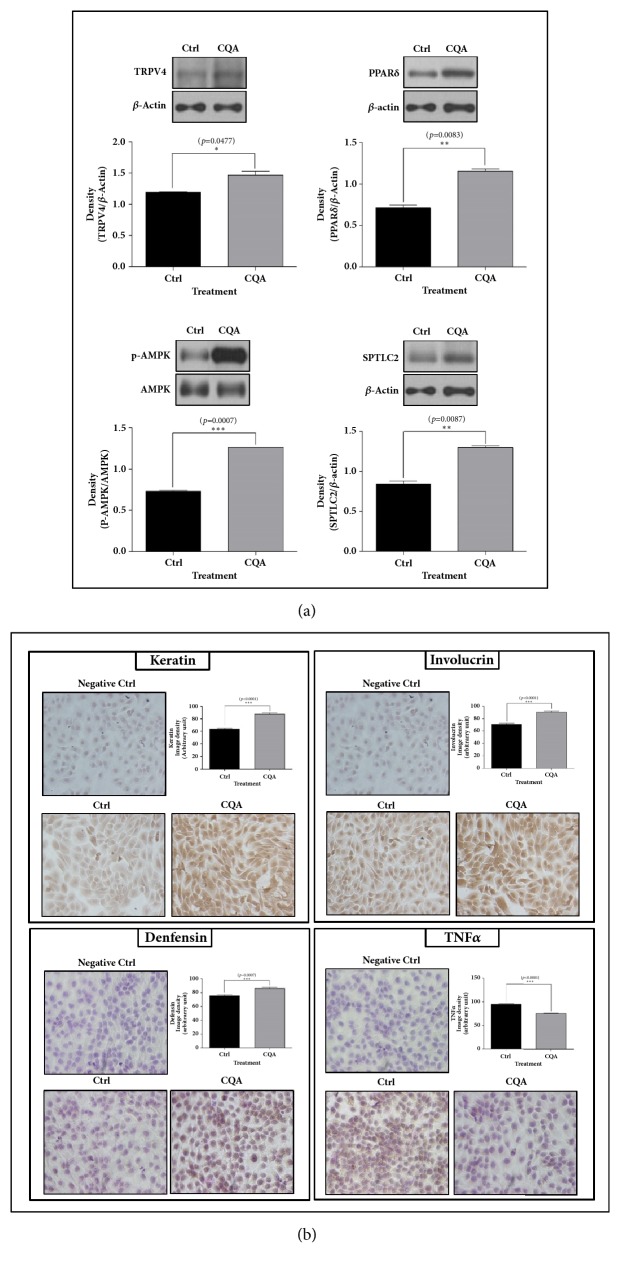
**Western blot analyses for TRPV4, AMPK, PPAR** **δ** **, and SPTLC2, and ICC for keratin, involucrin, defensin** **β** **1, and TNF** **α** **in HaCaT cells treated with caffeoylquinic acid.** CQA increased protein levels for TRPV4, PPAR*δ*, AMPK, SPTLC2, keratin, involucrin, and defensin*β*1 (a, b); however, it decreased TNF*α* expression (b). Images were taken at 200 × magnification. The results are expressed as means ± SEM. N=3 for each group. Values were statistically analyzed by unpaired* t*-test. All experiments were repeated over three times.

3, 5-DCQA as the most abundant caffeoylquinic acid constituent of AG extract increased the protein levels for TRPV4, PPAR*δ*, AMPK, SPTLC2, keratin, involucrin, and defensin*β*1 likewise the AG ethyl acetate extract in HaCaT cells (Figures 7(a) and 7(b)). But TNF*α* expression related to skin inflammation was decreased by the substance (Figure 7(b)).

### 3.7. Aster glehni Extract and 3, 5-DCQA Increased the Number and AMPK Protein Level of HaCaT Cells. However, They Did Not Affect the Apoptotic Marker, cPARP. AG Did Not Significantly Affect AMPK*α*1 mRNA Level

The treatments of AG extract and 3, 5-DCQA increased the proliferation of HaCaT cells (Additional Figure 1A); also they elevated the total AMPK protein level (Additional Figure 1B). AG and 3, 5-DCQA did not induce the apoptosis in HaCaT cells (Additional Figure 1B). Additionally, AG treatment did not significantly affect the mRNA expression of AMPK*α*1 in HaCaT cells (Additional Figure 2).

## 4. Discussion

PPAR*δ* as a nuclear receptor alleviates metabolic diseases such as obesity and atherosclerosis [27, 28]. PPAR*δ* participates in skin barrier protection via the following mechanisms. PPAR*δ* ligand treatment stimulates stratum corneum formation and permeability barrier development in a fetal rat explant culture model [13]. In addition, the epidermal integrity is disrupted and inflammation is increased in PPAR*δ* knockout mice [14]. Generally, AMPK is involved in decreasing inflammation as well as ameliorating the symptoms of metabolic diseases, which is also regulated by PPAR*δ* [20, 29]. Activated AMPK suppresses the increase of matrix metalloprotenase1 induced by ultraviolet radiation in HaCaT cells [15]. Moreover, in human keratinocytes, AMPK is involved in autophagy regulation through the inhibition of mTOR signal pathway by apigenin [30].

In this study, SDS- and DNCB-induced decreases in protein expression of keratin, involucrin, and defensin*β*1 were recovered by AG extract. In addition, SDS- and DNCB-induced increases in expression of TNF-*α* were normalized by AG treatment. The levels for PPAR*δ* and AMPK were elevated by AG extract treatment compared to SDS or DNCB treatment alone. Furthermore, the positive effects of AG extract were offset by PPAR*δ* antagonist, GSK0660; therefore, we hypothesized that the effects of AG on keratinocytes are dependent on PPAR*δ*. In the condition of AG and DNCB or AG and SDS treatment, the pattern of AMPK protein expression was different from the mRNA expression of AMPK in normal condition. From the data on AMPK expression, we can suppose that the AMPK protein expression by AG at pathological conditions may be attributed to the translational modification or stability of protein. Besides PPAR*δ* and AMPK, TRPV4 also plays important role in the maintenance or protection of skin permeability barrier. TRPV4 is generally known as a calcium ion (Ca^++^)-permeable cation transporter, and it responds to mechanical stress such as swelling [31]. In dermatology, it protects the skin permeability barrier via strengthening of tight junctions [16, 17] and increased keratin synthesis in HaCaT cells treated with baicalein [32]. The role of TRPV4 as a Ca^++^ transporter suggests that it participates in calcium signal transduction in keratinocytes. Ca^++^ generally functions as a signaling molecule in cells and stimulates the expression of biomarkers such as keratin1/10, involucrin, loricrin, and transglutaminase 1 involved in keratinocyte differentiation [33]. Moreover, Ca^++^ facilitates the transition of profilaggrin to filaggrin [34]. When the skin permeability barrier is disrupted, Ca++ gradient is lost. Subsequently, the expression of loricrin, filaggrin, and involucrin is decreased [35]. In our study, TRPV4 protein level was elevated by AG extract compared to DNCB or SDS-treated group; furthermore, TRPV4 antagonist negatively affected the biomarkers involved in the maintenance of permeability barrier. Therefore, from these results, we can suppose that TRPV4 is involved in the protection of skin permeability barrier, and it functions as a regulator in the action of AG extract. Furthermore, the results acquired from HaCaT cells treated by antagonists and siRNAs for PPAR*δ*, AMPK, and TRPV4 suggest that AG extract can protect keratinocytes through the sequential regulation of TRPV4→ PPAR*δ*→ AMPK pathway.

The 3,5-dicaffeoylquinic acid, the most plentiful substance in seven caffeoylquinic compounds of AG extract, showed equivalent effects with the AG ethyl acetate extract in the expressions of biomarkers related to the integrity and inflammation of permeability barrier in HaCaT cells. Therefore, we can suggest that the function of AG extract is attributed to caffeoylquinic acids.

Moreover, when AG or CQA treated HaCaT cells, the cell number was increased. Therefore, it is supposed that one of protecting mechanisms of AG on keratinocytes may be the stimulation of cell proliferation (additional Figure 1A).

It is generally known that atopic dermatitis is incurred through various causes such as the impairment of skin permeability barrier by irritant, allergen, and other external materials, genetic defects, immunological imbalance, and environmental pollution and it is easily recurred [36, 37]. Remedies for atopic dermatitis include steroids, antihistamines, and immunomodulators. But, the need for more effective new drugs having less side effects compared to existing medicines is very high now.

Therefore, this study may be a small stepping stone in the development of drugs for contact dermatitis and atopic dermatitis.

This study had done the effects and the mechanism of total AG extract and 3,5-dicaffeoylquinic acid in only* in vitro* experiments, so, in our further study, animal study and experiments for other caffeoylquinic acids should be additionally done to prove more precise mechanisms of AG.

**Figure 8:**
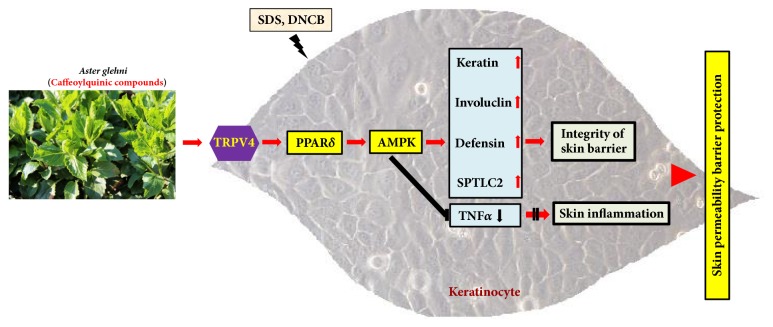
**Schematic diagram for the functional mechanism of* Aster glehni *extract in keratinocytes.** The effects of* Aster glehni* on skin barrier protection are mainly mediated via the sequential regulation of TRPV4→ PPAR*δ*→ p-AMPK→ skin barrier constituting elements; also it is partly done by anti-inflammation through the inhibition of TNF*α*. Meaning of symbols: arrow means activation, up and horizontal line means inhibition, and double vertical line means blocking.

Consequently, our study suggests that caffeoylquinic compound-rich AG extract can protect the permeability barrier through the sequential regulation of TRPV4-PPAR*δ*-AMPK pathway in human keratinocytes HaCaT cells treated with SDS or DNCB (Figure 8).

## Data Availability

We can provide the data when there is a request from other researcher (data available on request). The e-mail addresses for the data request are as follows: mdhsseo@unitel.co.kr and lyj2333@hanmail.net.

## Supplementary Materials

Supplementary Materials Supplementary Figure 1: cell number profile and protein levels for AMPK and cPARP in HaCaT cells treated with AG or CQA. Supplementary Figure 2: RT-PCR for AMPK in HaCaT cells treated with AG.
