# Supplementary material for: Aster glehni Extract Containing Caffeoylquinic Compounds Protects Human Keratinocytes through the TRPV4-PPARδ-AMPK Pathway
Source: Evid Based Complement Alternat Med. 2018 Dec 9;2018:9616574. doi: 10.1155/2018/9616574 (PMC6304624; doi:10.1155/2018/9616574)

Supplementary figure legends

Additional Fig. 1 Cell number profile and protein levels for AMPK and cPARP in HaCaT cells treated with AG or CQA.

The cell number of HaCaT cells was increased by the treatment of AG or CQA (A). The AMPK protein level was significantly increased by AG treatment, and in the case of CQA treatment, an increasing trend was observed (B). cPARP (an apoptotic marker) protein level was not changed by the treatment of AG or CQA (B). The results are expressed as means ± SEM. N=3 for each group. Values were statistically analyzed by unpaired *t*-test and one way ANOVA. The dash-dotted line means the one way ANOVA result between ctrl and other groups. All experiments were repeated over three times.

Additional Fig. 2 RT-PCR for AMPK in HaCaT cells treated with AG.

The mRNA level for AMPK in AG treatment group showed an only increased tendency compared with control group. The results are expressed as means ± SEM. N=3 for each group. Values were statistically analyzed by unpaired *t*-test. All experiments were repeated over three times.

Additional Fig. 1

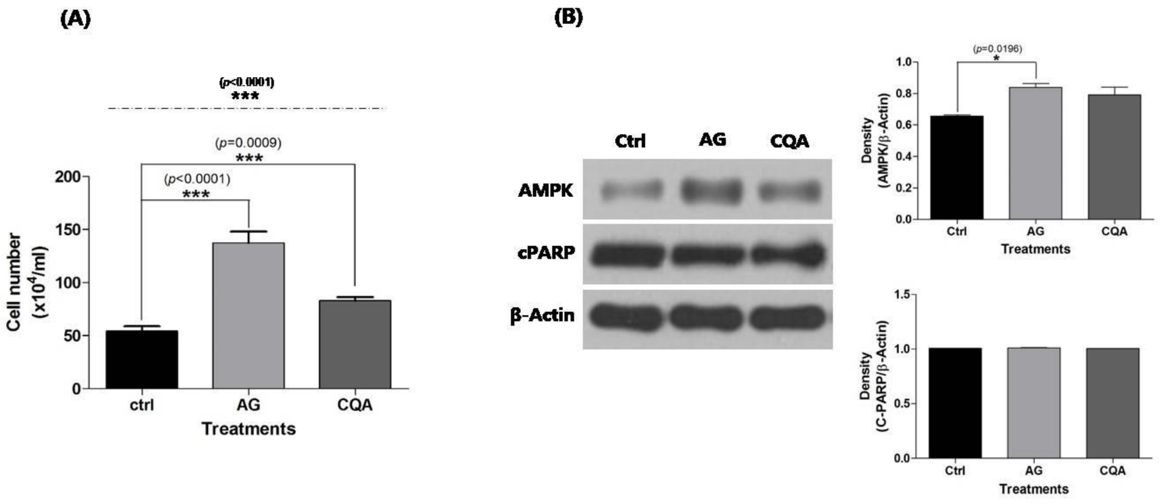

Additional Fig. 2

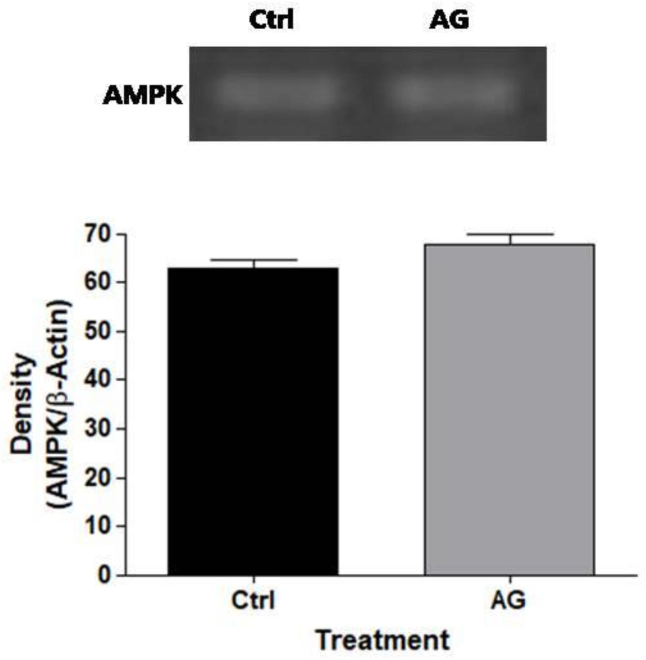

Supplement: Supplementary Materials — Supplementary Figure 1: cell number profile and protein levels for AMPK and cPARP in HaCaT cells treated with AG or CQA. Supplementary Figure 2: RT-PCR for AMPK in HaCaT cells treated with AG. [file 9616574.f1.zip › mat.9616574.v3.pdf]
