# Supplementary figures and images for: Aster glehni Extract Containing Caffeoylquinic Compounds Protects Human Keratinocytes through the TRPV4-PPARδ-AMPK Pathway
Source: Evid Based Complement Alternat Med. 2018 Dec 9;2018:9616574. doi: 10.1155/2018/9616574 (PMC6304624; doi:10.1155/2018/9616574)

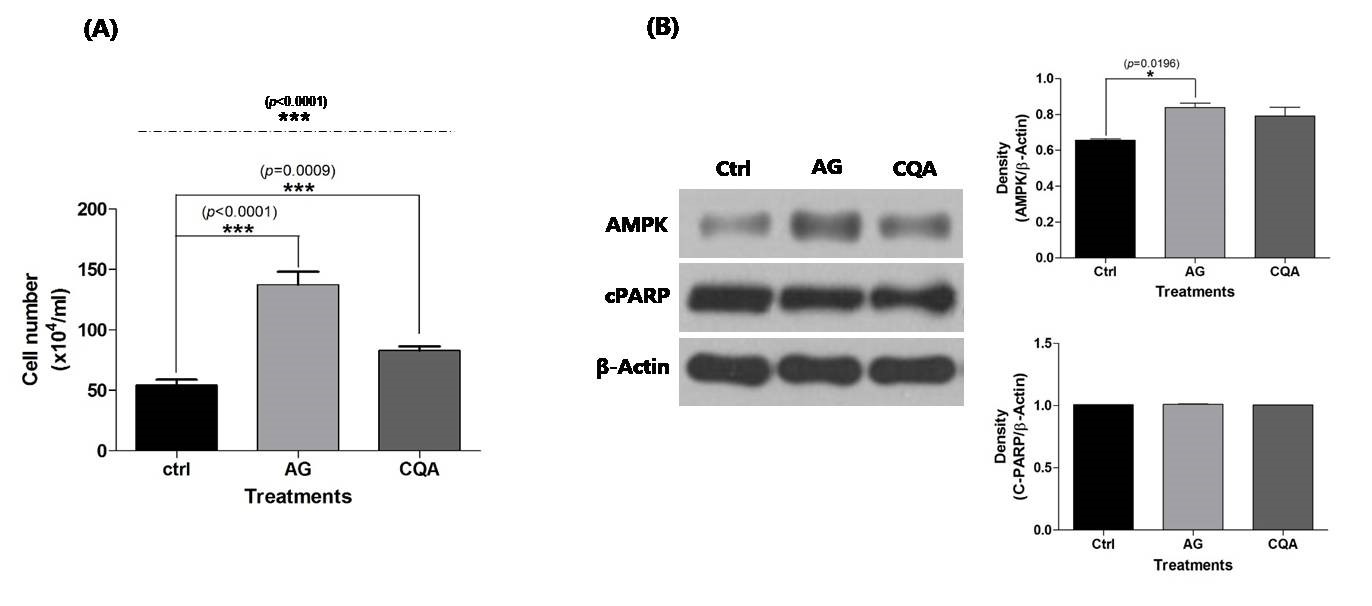

Supplement: Supplementary Materials — Supplementary Figure 1: cell number profile and protein levels for AMPK and cPARP in HaCaT cells treated with AG or CQA. Supplementary Figure 2: RT-PCR for AMPK in HaCaT cells treated with AG. [file 9616574.f1.zip › Supplementary Fig. 1_ECAM_2593461.jpg]

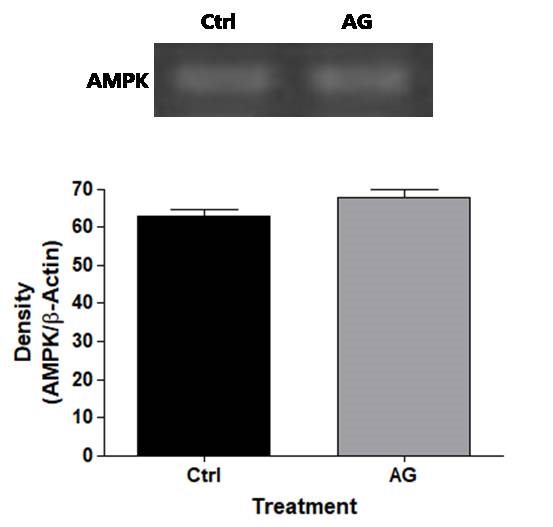

Supplement: Supplementary Materials — Supplementary Figure 1: cell number profile and protein levels for AMPK and cPARP in HaCaT cells treated with AG or CQA. Supplementary Figure 2: RT-PCR for AMPK in HaCaT cells treated with AG. [file 9616574.f1.zip › Supplementary Fig. 2_ECAM_2593462.jpg]
